# Machine learning-based prediction of glioma margin from 5-ALA induced PpIX fluorescence spectroscopy

**DOI:** 10.1038/s41598-020-58299-7

**Published:** 2020-01-29

**Authors:** Pierre Leclerc, Cedric Ray, Laurent Mahieu-Williame, Laure Alston, Carole Frindel, Pierre-François Brevet, David Meyronet, Jacques Guyotat, Bruno Montcel, David Rousseau

**Affiliations:** 1 Univ Lyon, Université Claude Bernard Lyon 1, CNRS, Institut Lumière Matière, F-69622, Villeurbanne, France, 10 Rue Ada Byron, 69622 Villeurbanne, France; 2 CREATIS, Univ Lyon, CNRS UMR5220, INSERM U1044, Université Claude Bernard Lyon1, INSA Lyon, Villeurbanne, France; 3 Hospices Civils de Lyon, Centre de Pathologie et de Neuropathologie Est, Lyon, F-69003 France; 4 Cancer Research Centre of Lyon, Univ Lyon, INSERM U1052, CNRS UMR5286, Lyon, France, Université Claude Bernard Lyon 1, Lyon, France; 5 Laboratoire Angevin de Recherche en Ingénierie des Systèmes, UMR INRA IRHS, Université d’Angers, 62 avenue Notre Dame du Lac, 49000 Angers, France

**Keywords:** Diagnostic markers, Surgical oncology

## Abstract

Gliomas are infiltrative brain tumors with a margin difficult to identify. 5-ALA induced PpIX fluorescence measurements are a clinical standard, but expert-based classification models still lack sensitivity and specificity. Here a fully automatic clustering method is proposed to discriminate glioma margin. This is obtained from spectroscopic fluorescent measurements acquired with a recently introduced intraoperative set up. We describe a data-driven selection of best spectral features and show how this improves results of margin prediction from healthy tissue by comparison with the standard biomarker-based prediction. This pilot study based on 10 patients and 50 samples shows promising results with a best performance of 77% of accuracy in healthy tissue prediction from margin tissue.

## Introduction

Gliomas account for more than fifty percent of primitive brain tumors. They are infiltrative tumors, with a margin difficult to identify and discriminate from the surrounding healthy tissues. The world health organization (WHO) classifies gliomas in 4 grades^1^, but most studies commonly consider two separate groups: High-Grade Gliomas (HGG) and Low-Grade Gliomas (LGG). Studies have shown that in 85% cases, recurrences of HGG are localized less than 2 centimeters away from the initial tumor^2^. Then, improving the extent of resection is relevant to prevent recurrence and improve life quality and expectancy^3–5^. Pre-operative MRI combined with neuro-navigation is currently used in the operating theater^6,7^ but shows strong limitations^8–10^. 5-aminolevulinic acid (5-ALA) induced protoporphyrin IX (PpIX) fluorescence microscopy has shown its relevance in neuro-oncology^11^. PpIX absorbs light at 405 nm and emits fluorescence with a main peak centered at 634 nm. This technique is the actual clinical standard for PpIX-based surgical assistance. However, its sensitivity is still limited when applied to low-density infiltrative parts of HGG^12,13^ or to LGG^14^.

Various 5-ALA induce PpIX fluorescence spectroscopy methods have been proposed to overcome these sensitivity issues. Previous works^6,15–24^, focus on the extraction of biomarkers from the measurements, based on a priori information on the link between the biomarkers and the microenvironment of PpIX. These approaches are known as expert-based, and various biomarker models have been proposed in the literature. Quantification of PpIX concentration^15^ show enhanced sensitivity either in HGG^16^ or in LGG^17^. Normalization procedures of biomarkers can also increase their robustness^6,18,19^. Other works suggest that relevant models could be obtained based on the shape of the PpIX emission spectrum^18–26^. These works show that the PpIX fluorescence emission spectral complexity in tissue is closely linked with the pathological status. However, the still unsolved origin of this complexity impairs the extraction of the best features with an expert-based related method, thus preventing the classification of measurements into relevant pathological status.

**Figure 1:**
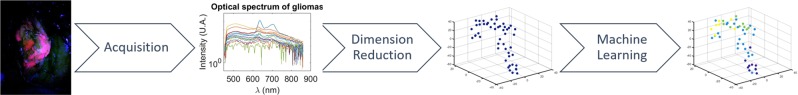
Global view of the proposed machine learning-based prediction of glioma margin by PpIX fluorescence spectroscopic measurements. In this study, the data set is composed of 50 samples from 10 patients. From left to right, the optical spectrum of cells around a tumor is measured. The dimension of the spectral information is then reduced to lower the redundancy. Supervised or unsupervised algorithms are finally used to classify the data and create a prediction of tissue state from the PpIX fluorescence spectroscopic measurements.

In this study, we adopt a different approach for the prediction of glioma margin from fluorescence information. Instead of choosing a small amount of numerical features used as biomarkers like in the recent above-cited literature^6,15–24^, we propose to investigate the prediction of glioma margin with an entirely data-driven approach where no assumption of feature selection based on expert is made. To this purpose, we implement, for the first time to our knowledge in this context, a machine learning classification approach. The pipeline, as illustrated in Fig. 1 and detailed in the material and method section predicts glioma margin from the raw fluorescence spectrum. This is done on the same data acquired previously in a surgical procedure of 5-ALA induced PpIX guided glioma removal and which had been only processed so far with a biomarker approach^23^. This choice enables a comparison of the prediction performance of glioma margin from a biomarker approach with a novel expert-independent point of view. Also, as another element of novelty, the prediction of glioma margin is performed from 3 different fluorescent spectra in response to 3 distinct excitation wavelengths taken successively over the same area, while previously only a few features extracted from a single fluorescent spectrum were used for analysis^23^.

## Results

Before comparing performances of supervised and unsupervised classification of machine-learning-based with an expert-based approach, we provide the estimation of the number of clusters and the identification of the best spectral features with the pipeline of Fig. 1 as described step-by-step in the Method section.

### Data driven estimation of the number of clusters corresponds to the clinical taxonomy

**Figure 2:**
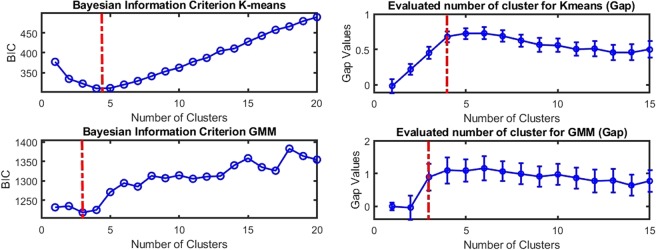
Bayesian inference criterion (BIC) (left) and gap criterion (right) as a function of the number of clusters for K-means (top row) and GMM (bottom row). The minimum of the BIC and maximum of the gap criterion (highlighted in the red dash-dotted line) correspond to the optimal number of clusters in our data. Interestingly K-means and GMM are best described with 3 or 4 clusters which fit with the red dotted lines corresponding to the number of classes from the clinical taxonomy.

The number of classes in the data set was automatically estimated using data-driven methods. As illustrated in Fig. 2, the optimum number of clusters is robustly found between 4 and 5. This is recorded by the Bayesian information criterion (BIC)^27^ and gap criteria^28^ where the extremum of the curves indicate the optimal number of clusters for both tested clustering methods (K-Means, Gaussian Mixture Models). Interestingly, while obtained here from a purely data-driven approach, this number of clusters is compatible (see red dotted lines in Fig. 2) with the number of classes proposed independently by the clinical taxonomy described in^23^ from histological images: tumor core, high-density margin, low-density margin, healthy tissue.

### Identification of best spectral features

**Figure 3:**
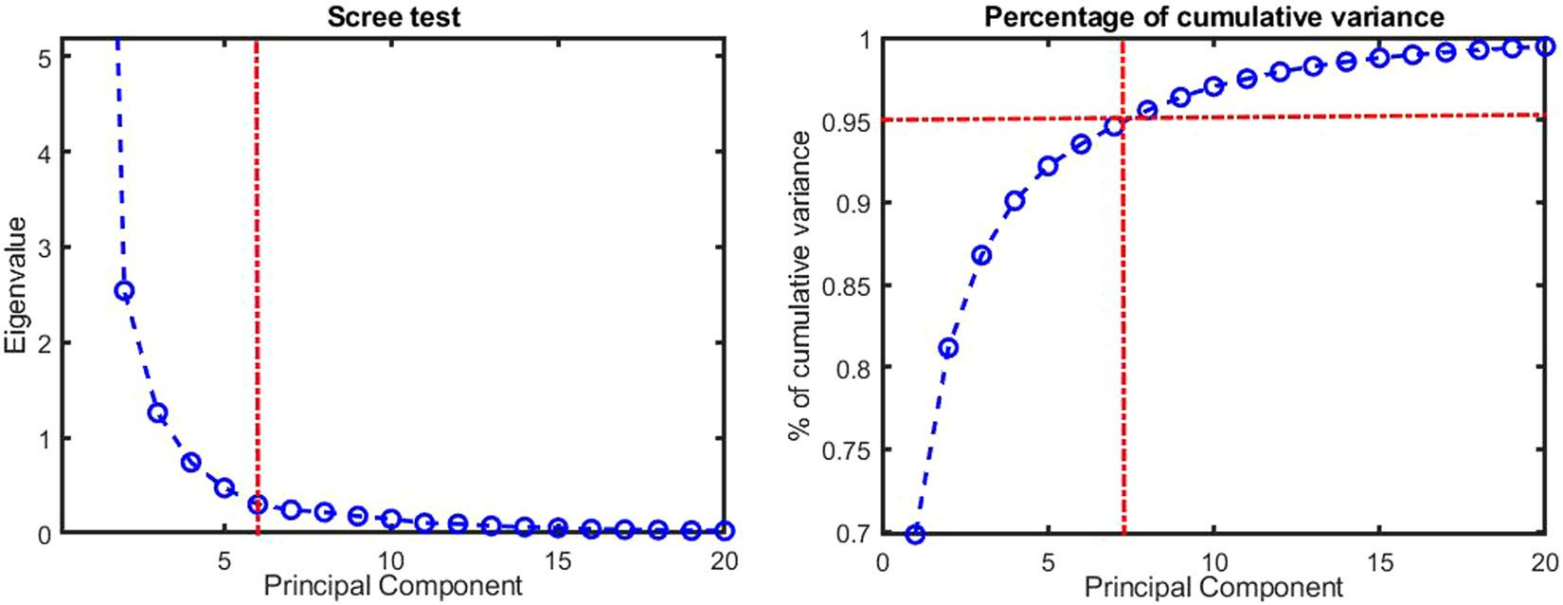
Scree test (on the left) and cumulative variance (on the right) of principal component analysis. A minimum of 5 principal components is required to describe the data variance as can be inferred from the Scree test “elbow” and the saturation around 95% of the cumulative variance highlighted in red dotted lines.

**Figure 4:**
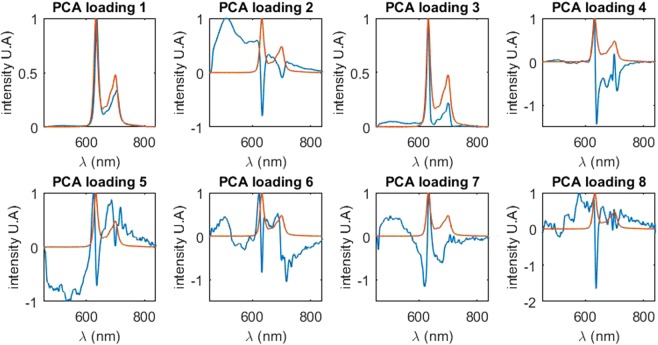
Normalized principal component of the PCA (in blue) in the original feature space (*i.e.* the optical spectrum space). For each principal component, the reference spectrum of the PpIX (for the state peaking at 634 nm) is also plotted (in red). For better comprehension, only one of the three fluorescence emission spectrum from the original feature space is represented as they are similar for all three excitation wavelengths. The first principal component is similar to the spectrum of the PpIX with a peak of 636 *nm*. The second principal component is best described as the autofluorescence of the measured tissue, *i.e.* the contribution of other fluorophores. The five following components all show a peak shifting between 620 *nm* and 636 *nm*.

Our original feature space composed of raw fluorescence spectrum with 900 emission wavelengths (from 435 to 840 *nm*) taken at three distinct excitation wavelengths includes 2700 features. In order to both select the best spectral features and analyze their shapes, dimension reduction techniques were applied. Based on the principal component analysis (PCA)^29^, the Scree test^30^ and the cumulative variance were used to find the statistically relevant principal spectral components. As illustrated in Fig. 3, either the Scree test (left) and the cumulative variance (right) in the PCA analysis show that a minimum of 5 components is required to describe the data variance. Indeed, the Scree test “elbow” and the saturation around 95% of cumulative variance are both found around five components (given by the red dotted vertical line in Fig. 3). By comparison, in our previous study^23^, for one excitation wavelength, 2 features were extracted from the spectrum: the relative intensity of the component leading to a peak of PpIX fluorescence at 620 *nm* (PpIX620) and the relative intensity of the component leading to a peak PpIX of fluorescence at 634 *nm* (PpIX634). With three excitation wavelengths, the resulting feature space of this model is 2 × 3 = 6 descriptors to describe the variability of the data. Thus the number of statistically relevant descriptors computed with the data-driven approach with the principal components analysis (5 components) is compatible with the expert-based model (6 components). In addition to the number of relevant principal components, the shapes of these components were analyzed in the original feature space, ***i.e****.* the fluorescence emission spectrum. These are plotted in Fig. 4. In order to simplify the visualization, only the result for one excitation wavelength (405 *nm*) is displayed. However, results were very similar to the two other excitation wavelengths.

It is then interesting to compare the shape of the principal components extracted from the data-driven PCA approach with the spectral patterns extracted in the expert-based approach of ^23^, as shown in Fig. 4. Not surprisingly, the first principal component is very similar to the fluorescence emission spectrum of the PpIX^19^ with a peak around 634 *nm* and, also not surprisingly the second component corresponds to the autofluorescence of the tissue, which can be linked to numerous endogenous autofluorescent molecule such as NADH, FAD, lipofuscin^31,32^. These molecules are also known to be correlated with cancerous pathological status through the Warburg effect^22,33^ which modify cell metabolism and favorize glyclolysis. The third principal component is a mix between the spectrum of PpIX with a peak around 632 *nm* and the fluorescence previously described. The fourth principal component is comprised of a peak at 629 *nm* and a smaller one at 695 *nm*. The fifth and last relevant principal component shows a maximum at 625 *nm*. All principal components present a peak varying from 622 *nm* (principal component 6) to 636 *nm* (principal component 1). Interestingly, the variance of our normalized spectra is best described as a blue-shift of the PpIX spectrum peak from 636 *nm* to 620 *nm*. Remarkably, the shape of these principal components extracted with a purely data-driven approach corresponds to spectral components identified in the expert-based studies^19,23^.

### Good predictions are obtained in unsupervised and supervised modes

**Figure 5:**
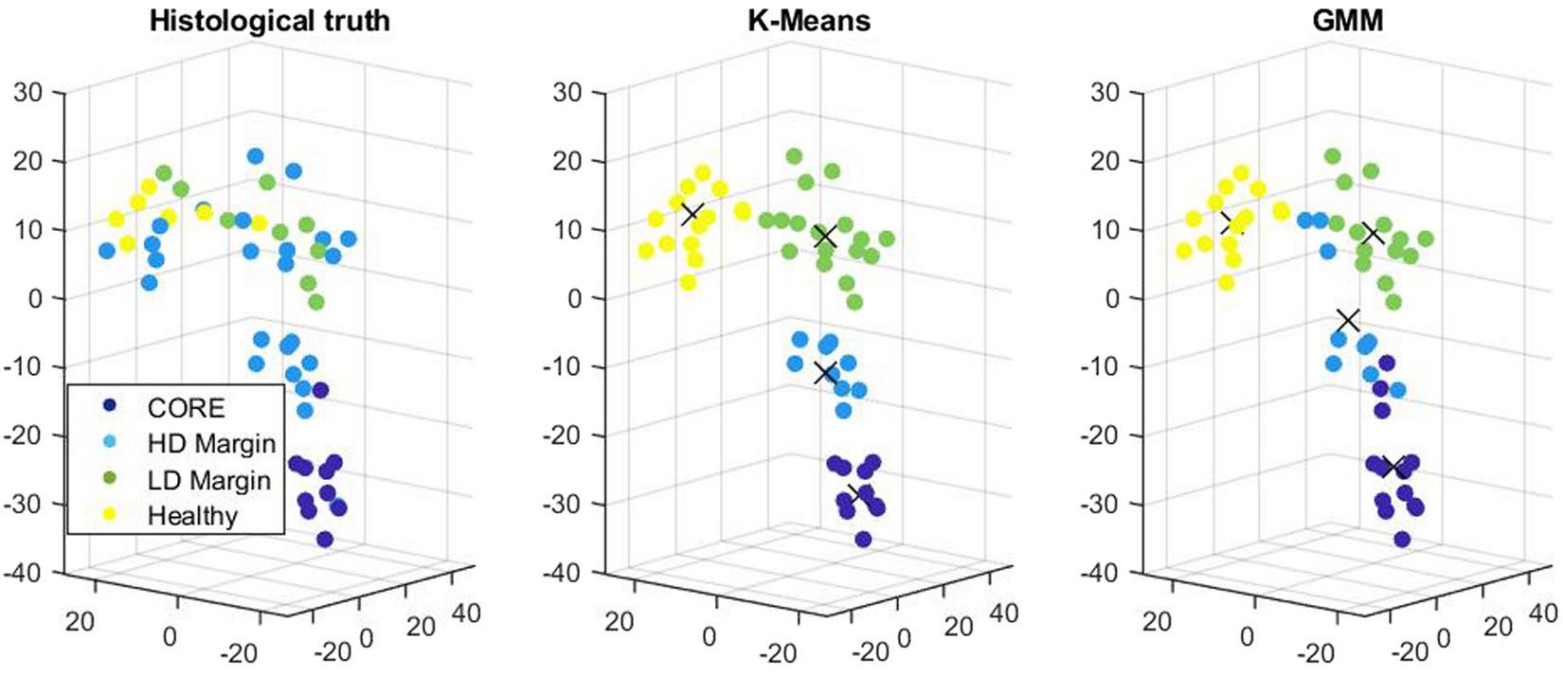
Unsupervised classification in a T-SNE reduced space. From left to right: the histological truth as given by the anatomopathologist, K-means classification and GMM classification both with 4 clusters.

Unsupervised learning was then used to find clusters among a reduced feature space autonomously. Two methods were tested, including PCA and T-SNE algorithms^34^. A dimension reduction to 3 was chosen to facilitate the visualization of clusters. With this choice, the resulting clustering presented a better accuracy with T-SNE rather than PCA, and thus only T-SNE results are presented here when applied to K-means and GMM for the four classes identified previously. As K-Means and GMM are randomly initiated, in order to assess the quality of our results, the classification was reproduced twenty times and averaged. Results can be seen for four classes with K-means in Table 1 when the entire feature space (3 excitation wavelengths) was considered. A comparison with a feature space based on each excitation wavelength was also plotted and discussed further in the Supplementary Material. The typical clustering results are displayed in Fig. 5.

**Table 1:** Confusion matrix for K-means with 4 classes: tumor core, high density margin, low density margin and healthy tissue.

	Predicted Core	Predicted HD Margin	Predicted LD Margin	Predicted Healthy
True Core (10)	8 (80%) *σ* = 0	1.9 (19%) *σ* = 0.31	0 (0%) *σ* = 0	0.1 (1%) *σ* = 0.31
True HD Margin (24)	1.3 (5%) *σ* = 0.5	7.1 (30%) *σ* = 0.32	9.7 (40%) *σ* = 0.48	5.9 (25%) *σ* = 0.32
True LD Margin (9)	0 (0%) *σ* = 0	0.1 (1%) *σ* = 0.31	5.6 (62%) *σ* = 0.52	3.3 (37%) *σ* = 0.48
True Healthy (7)	0 (0%) *σ* = 0	0 (0%) *σ* = 0	1 (14%) *σ* = 0	6 (86%) *σ* = 0

Statistics (average, standard deviation) result from 20 predictions. In each cell, from left to right: number of instances, percentage of total class population and standard deviation.

As seen in Fig. 5, the four resulting computed clusters fairly correspond to the clinical taxonomy given by the anatomopathologist (on the left). In addition, these clusters are in this feature space aligned along with the ordinal severity of symptoms following the density of diseased cells with maximization of the distance between tumor core and healthy cells. Both K-means and GMM methods (middle and right of Fig. 5, respectively) give results in accordance with the histological truth. Figure 5 and Table 1 constitutes promising results. While accurate prediction for the tumor core is logical since it is the main target of the PpIX, we also see that accurate prediction is obtained for the healthy tissue. Overall a prediction between healthy tissue, tumor core, and margin led to a 73% accuracy and went up to 77% for classification with only three classes. To further stress the interest of machine learning-based prediction of glioma margin, a supervised method was also used. Typical results are shown in the Supplementary Material.

### Comparison between machine-learning-based and expert-based feature selection

**Figure 6:**
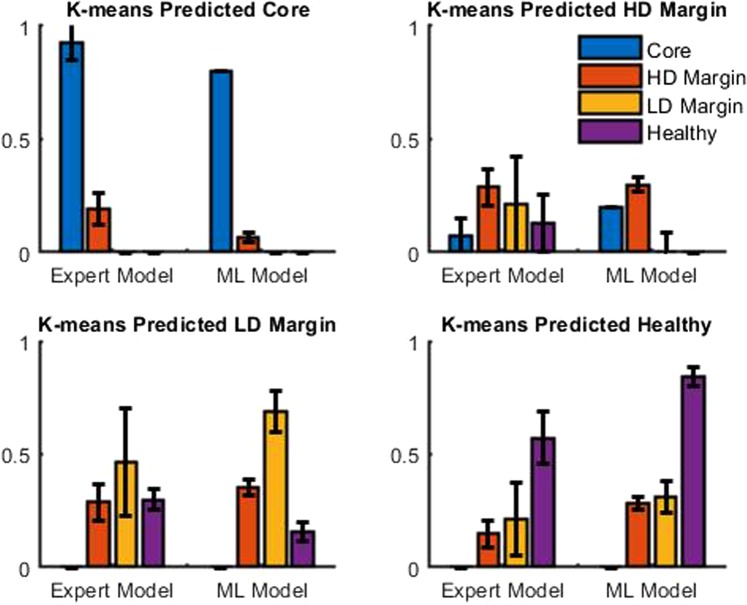
Comparison of confusion matrix for K-means with 4 classes: tumor core, high and low density margin and healthy tissue. Average result of 20 predictions. ML model is Machine learning-based Model, HD stands for high density and LD for low density.

As stated above, the number of clusters and the shape of the principal component calculated are remarkably similar to what has been described in^23^. The benefits of this fully automated approach compared to the expert-based model is thus investigated. We use the expert-based method of our previous study^23^. This consist in a fitting process of the fluorescence spectrum to retrieve the relative contribution of the two states of PpIX, PpIX620 and PpIX634 for each excitation wavelength. In this study this process is led for the three excitation wavelength, instead of only at the 405 *nm* excitation wavelength in the previous study. The resulting feature space of dimension 6 is then normalized and reduced using T-SNE to 3 dimensions like with the machine learning approach in order to enable a fair comparison between the discrimination power of each method. The average result of 20 predictions can be seen in Fig. 6. The machine learning-based method shows a clear superiority over the expert-based model. In particular, the “Healthy tissue” case is significantly better for true positive and true negative while being comparable or better for false positive.

This result demonstrates that despite similar features, the fully automated machine-learning-based method shows significantly better results than the previously used expert-based model. In the expert-based model of ^23^, the fit was between 585 and 640 nm, which did not include patterns around 700 nm which shows variability among the datasets. Also, in^23^ the autofluorescence was used to normalize every spectrum and was then substrated from the signal. In our case, autofluorescence was used differently since it was kept in the signal (mainly present in the second component of the PCA in Fig. 4).

## Discussion

This study demonstrated the interest of a machine learning-based approach for the prediction of glioma margin. This approach found spectral features important for the prediction of glioma margin, which happens to be close to the one selected in the expert-based model of ^23^. This is an important result since this is obtained by two independent ways on the same dataset. It reinforced, quantitatively, the evidence of the interest in using the blue-shift in the PpIX fluorescence spectrum as a mean of discrimination between margin and healthy tissue. Other works already suggested a wavelength blue-shift of the peak intensity of the emission spectrum correlated with the tissue pathological status^18,19,23^. In particular, a second peak of fluorescence at 620 nm has been observed in tissues^19–23,25,26^ or in cell culture^21,24^. It is *in vivo* origin is still an open issue, some works supporting the assumption that it is related to different aggregates of PpIX^19,23,24,26^, other works^20,35^ argue that it is a fluorescence induced by the precursors of PpIX, uroporphyrins or coproporphyrins. This work cannot give clues on the origin of the blue-shift. However, the relevance of the blue-shift effect is retrieved in this data-driven approach. This reinforces the expert-driven previous works^18–26^ focusing on the blue-shift. The biological mechanism behind this shift remains to be uncovered to this day and is not the subject of this work. In this section, we rather discuss some elements of data preparation, excitation wavelengths, and further machine learning approaches.

### Data sample preparation

By contrast with^23^, the distinction between LGG and HGG was not done to increase the sample size of clusters. This choice could be discussed since the pathology is classified as different in high grade and low grade from the perspective of histology, which is based on tissular structures at the supracellular scale. However, a posteriori, we found that this choice does not prevent a reasonably good clustering prediction for the whole data set. In a future study, with increased sample size, it would be interesting to compare the clustering with labels differentiating LGG and HGG and the fused approach followed in this study.

### Multi-excitation wavelengths

The guideline of the article is to investigate how the increase of the fluorescence feature space can contribute to improving the performance of classification by comparison with an approach based on few fitted parameters. We extend the feature space to the entire spectrum of a single excitation wavelength in the core of the manuscript. We demonstrated that this feature space extension produces a gain of classification performances. In the complementary material of the article, we also report the performance when the feature space is increased to an additional fluorescence spectrum obtained with other excitation wavelengths. In order to probe the two PpIX states (peaking at 634 *nm* and 620 *nm*), the optical fluorescence spectra were acquired for three different LEDs exposition: 385, 405 and 420 *nm*. We investigated the potential of using different excitation wavelengths to expand the feature space, suppress potential degeneracy, and potentially get more accurate information and better insight. Using a PCA, we analysed the principal component in the original feature space (the eigenvectors in the spectral space). However, the resulting PCA were rather similar for all 3 LEDs, not showing significantly different information with respect to the wavelength. This is probably due to the significant overlapping of the emission spectra of the 3 LEDs. Another explanation for this degeneracy can be that minor change in the spectrum acquired with different excitation wavelengths are not distinguished because the small sample size does not allow for a minor and subtle change in the spectrum to be discriminating in this study.

### Supervised learning

Supervised learning showed (in Supplementary Material Section) consistent results with unsupervised learning. This is an interesting result since it shows the robustness of the classification with various machine learning algorithms. While it is certainly encouraging, we cannot be definitive about these supervised methods until the sample size is increased. Such input of more data would allow a fine-tuning of these models or a more advanced supervised classifier (such as deep neural networks), including more hyperparameters. Extended cohort and larger training dataset would also enable to perform classification in real time for clinical use without the need to perform cross-validation.

## Conclusion

In this article, we have demonstrated the interest of a machine learning approach for the prediction of glioma margin from 5-ALA induced PpIX fluorescence spectroscopy. When considering the entire raw spectrum as input feature space, classical dimension reduction was shown to select spectral patterns similar to those identified around 634 *nm* and 620 *nm* as possible biomarkers for margin prediction in our previous expert based work^23^. This pure data-driven proof, independent from the expert-based approach found in the current literature, is significant since the biochemical or physical origin of these spectral biomarkers is not yet understood. A second interest of the machine learning approach proposed here is that it shows an increase of discrimination as compared to our previous expert-based features used as biomarkers^23^. The best performance of 77% of accuracy between healthy tissue and margin is found. Despite the relatively small size of the data set considered here, this can be considered as promising pilot results due to their self-consistency with the classical expert-based approach for feature selection. Repetition with a larger cohort will have to be carried out to establish the added value of the optical probe for surgery. With such an extended data set, other machine learning models with higher capability and tuning parameters could be tested. Other expert model^16,17^ performance could also be compare against data driven approach to test its robustness. Also, another direction of investigation for the future could be to enlarge the feature space. No positive effect on prediction performance was recorded when increasing the number of excitation with single-photon fluorescence in this study. Two-photons fluorescence or the effect of polarization could also be worth to investigate in this context while revisiting the proposed machine-learning approach.

## Methods

The data set was acquired during a clinical study led at the neurologic center of the Pierre Wertheimer hospital in Bron, France. This study was described in detail in previous works^23^. All experiments were in accordance and approved by the French Agency for Health (ANSM) and the local ethics committee of Lyon University Hospital (France). All participating patients signed written informed consent. Only a part of the acquired data was used since we focused on “*in vivo*” measurements and used the multi-wavelength excitation measurements. For readability purposes, we shortly described the method and added complementary information to the already published method of ^23^.

### Spectroscopic device

The developed device has been described in detail in previous works^23^. Here, as a novelty, multi-wavelengths excitation, not described previously, was used. Therefore, we describe here the setup, including the multi-wavelength excitation capabilities. Excitation was performed through three light-emitting diodes (LED) centered at 385 nm, 405 nm, and 420 nm with 7 nm Full-Width Half-Maximum (M385F1, M405F1, M420F1, Thorlabs). Emitted light was transmitted through three optical fibers (HCG M0600T, sedi-fibres) to a dedicated probe. The probe entrance consists of a bundle of 7 optical fibers of 600 *μ*m core diameter. The other ends of these fibers are cleaved, so that excited tissue area and emitting tissue area are the same. The light goes through a low-pass filter (Edmunds Optics OD4 low pass 450 nm) with a cutoff wavelength of 450 nm. This led to an output irradiance of 80 W/m, 30 W/m, and 50 W/m, respectively, for the LED centered at 385 nm, 405 nm, and 420 nm. Tissue reflectance was collected through the same probe, with a detection fiber, and goes through a high pass filter (HQ485LP, Chroma) with a cutoff wavelength of 485 nm. The filtered light was finally injected into a spectrometer (Maya2000, Ocean optics). Characterization of the system has been performed on calibrated phantoms^36^.

### Surgical procedure and data acquisition

Patients were given an oral dose of 20 mg/kg of body weight of 5 aminolevulinic acids (Gliolan; Medac GmBH) approximately 3 hours prior to the induction of anesthesia. For each patient, the standard surgical procedure started in order to expose the tissue, and, when asked by the surgeon, the surgical procedure was stopped so that fluorescence measurements were performed. Each acquisition was composed of 200 ms of duration, with the LED turned on, followed by the same duration with the LED turned off to get rid of ambient light coming from the operating room. For each measurement, 6, 12, and 6 acquisitions were led respectively for the LED centered at 385 nm, 405 nm, and 420 nm. This gave a total acquisition time of 9.6 s. The tissue was then removed and sent for histopathological analysis. These fluorescence measurements were performed in order to get different densities of infiltrative tumor cells per glioma. In total, 50 measurements were kept in this analysis.

### Histopathology

Histopathological analysis was performed on formalin-fixed paraffin-embedded biopsy tissue specimens processed for H & E staining. Each H & E stained tissue section was assessed for the presence of tumor cells, necrosis, mitotic activity, nuclear atypia, microvascular proliferation, and reactive astrocytosis. Molecular criteria were also assessed. Biopsy specimens were then classified into five categories based on WHO histopathological and molecular criteria^1^ as HGG solid part, HGG margin, HGG margin of low density, LGG and healthy tissue. Finally, LGG and HGG data were combined in order to increase the sample size and test the expert driven against data driven approaches. This clustering is supported by previous works^19^ showing that LGG and HGG margins share common properties in terms of PpIX fluorescence intensity. The samples from LGG patients were included in the healthy, HGG low-density margin, or HGG high-density margin depending on their pathological status. The resulting studied data set was composed of 50 samples from 10 patients. This includes 28 samples for HGG composed of 10 samples from tumor core, eight from the high-density margin, five from the low-density margin, and five healthy samples. Furthermore, this includes also 22 samples for LGG, 17 included in HGG high-density margin, three included in HGG low-density margin, and two included in healthy tissue, depending on their pathological status.

### Data processing pipeline

The data processing pipeline developed is illustrated in Fig. 1. In the first step, the spectrum was acquired with the optical system. Three fluorescence spectra corresponding to three excitation LED’s were acquired. The pathological status of the corresponding tissue was recorded from the histopathological analysis. In the second step, the spectrum was then normalized by the global energy of the spectrum. The feature spaces, created by the three spectra, being too large to be applied to clustering methods^37,38^ were reduced with dimension reduction techniques in a third step. In our case, principal component analysis (PCA) and t-distributed stochastic neighbor embedding (T-SNE), which are two basic techniques for dimension reduction, were tested^34,39^. PCA was mainly chosen among others, orthogonal transformations, for its exploratory ability to summarize data along with their main characteristics. T-SNE was mainly chosen for its ability to preserve local structure so that points close to one another in the high-dimensional feature space will tend to be close to one another in the reduced feature space. The number of principal components to retain is determined with the Scree test^30^ or by computing the number of principal components requested to reach 95% of cumulated variance. The last step corresponded to the classification from the reduced feature space in an unsupervised and supervised way. Two clustering methods were chosen for this study: K-Means^40^ and the Gaussian mixture model (GMM)^41^. A large variety of methods can be found in the literature with various levels of complexity and hyperparameters to be adjusted^42^. Because the size of the data set is fairly small, algorithms were chosen with a minimal amount of hyperparameters to be adjusted. K-Means cluster points in the feature space inside hyper-spheres according to a euclidian distance while GMM includes an additional degree of freedom in the organization of the points which are clustered inside hyper-ellispoïds. In K-Means and GMM, the number of clusters is a hyperparameter, which was determined with the Bayesian inference criterion^27^ and the “gap” criteria^28^. We compared the predictive value of the pure data-driven feature space with the predictive value of expert-based feature space. The expert-based feature space consists of two features *α*_634_ and *α*_620_ computed from the raw spectrum. As described in^23^, the other intrinsic fluorophores (at the exclusion of PpIX) emitting below 620 nm were removed in the emission spectrum. The resulting spectrum was then fitted with the contribution of two PpIX spectra acquired *in vitro*. One of those spectrums presented a peak assumed Gaussian at 620 nm, and its relative contribution to the resulting spectrum was named *α*_620_. The other one presented a peak also assumed Gaussian at 634 nm, and its relative contribution was named *α*_634_. An extensively detailed explanation of this feature space can be found in previous work^23^. The reference spectra in Fig. 4 was also taken from this previous work.

## Supplementary information

Supplementary Information 

## Data Availability

The datasets analysed during the current study are available from the corresponding author on reasonable request.
