# Supplementary material for: Machine learning-based prediction of glioma margin from 5-ALA induced PpIX fluorescence spectroscopy
Source: Sci Rep. 2020 Jan 29;10:1462. doi: 10.1038/s41598-020-58299-7 (PMC6989497; doi:10.1038/s41598-020-58299-7)
Supplement: Supplementary file 1 — Supplementary Information [file 41598_2020_58299_MOESM1_ESM.pdf]

Machine learning-based prediction of glioma margin from 5-ALA induced PpIX fluorescence spectroscopy

Pierre Leclerc[1,2], Cedric Ray[1], Laurent Mahieu-Williame[2], Laure Alston[2], Carole Frindel[2], Pierre-François Brevet[1], David Meyronet[3,4], Jacques Guyotat[3], Bruno Montcel[2,*,+], and David Rousseau[2,5,+]

[1]Institut Lumière Matière, ILM UMR CNRS 5306, Université Claude Bernard Lyon 1, Campus LyonTech La Doua, 10 Rue Ada Byron, 69622 Villeurbanne, France
[2]CREATIS, Université de Lyon, Université Lyon1, CNRS UMR5220, INSERM U1044, INSA Lyon, Villeurbanne, France
[3]Hospices Civils de Lyon, Centre de Pathologie et de Neuropathologie Est, Lyon, F-69003, France
[4]Cancer Research Centre of Lyon, INSERM U1052, CNRS UMR5286, Lyon, France, Université Claude Bernard Lyon 1, Lyon, France.
[5]Laboratoire Angevin de Recherche en Ingénierie des Systèmes, UMR INRA IRHS, Université d'Angers, 62 avenue Notre Dame du Lac, 49000 Angers, France
[*]Corresponding author: bruno.montcel@univ-lyon1.fr
[+]Equal contribution

ABSTRACT

supplementary materials.

Supplementary Data

Confusion matrix of K-means and GMM for each wavelength.

Different feature spaces including the fluorescence emission spectrum in response to the three different excitation wavelengths were compared for the prediction of glioma margin with K-means and GMM after dimension reduction. Results for comparison of different excitation wavelengths can be seen in Fig. 1. These results records no evidence of added value in using an excitation wavelength over one nor to combine them.

Supervised learning

As a complement to the unsupervised classifiers (K-means and GMM) tested in the core of the manuscript for glioma prediction we also assessed the performance of a basic supervised method. In order to create a robust statistic around the size-wise limited data sample, the classifier was train on a data set with randomize case order. This "new" classifier was train and tested using a 5-folds cross-validation method[?]. The whole process was repeated 50 times and a resulting confusion matrix was averaged. K-Nearest Neighbours (K-NN) was used[?]. K-NN is among the simplest supervised machine learning classification algorithms and makes no assumption about the data. Despite the small size of our data set, interesting results were achieved and can be seen in Fig. 2. While prospective, these results further demonstrates that using the fluorescence emission spectrum of PpIX for the classification of tissue states of gliomas is robustly found to be useful.

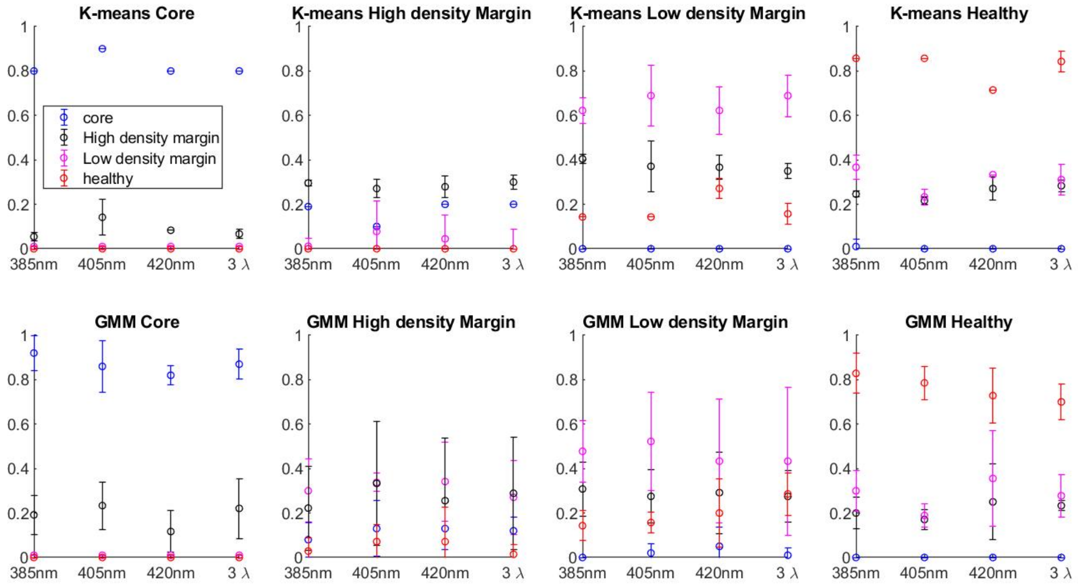

Figure 1. Representation of the confusion matrices of K-means and GMM for feature spaces based on the fluorescent emission spectrum of each individual wavelength or the three fluorescence emission wavelengths combined. Values plotted are the mean and standard deviation of 10 K-Means or GMM distribution. Each plot is the distribution of true histological classes for each predicted class (core, margin of high and low density and healthy). Top row stands for confusion matrix for K-means and bottom row for confusion matrix of GMM.

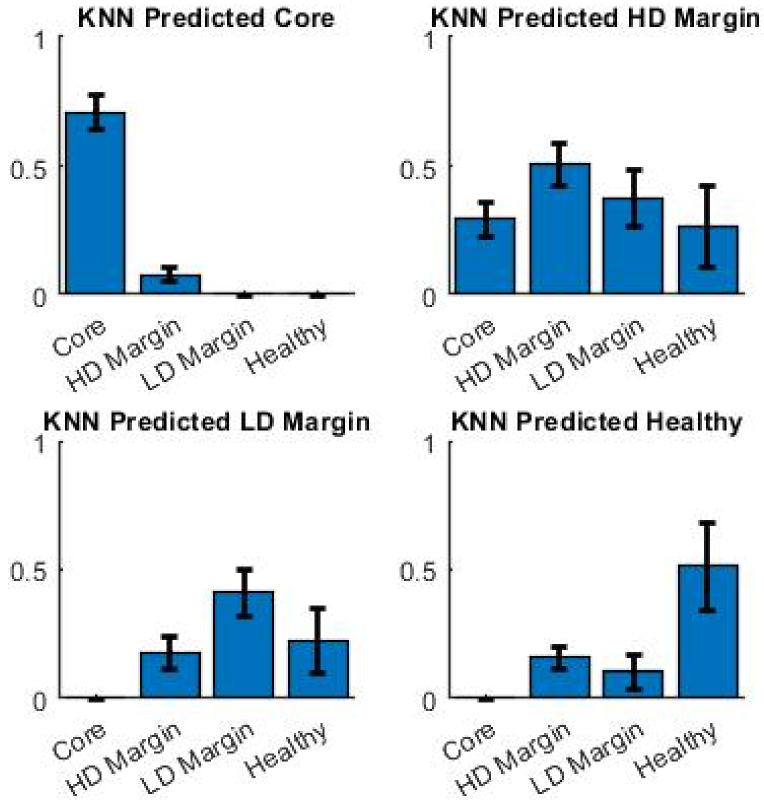

]

Figure 2. Results for a trained and tested K-NN classifier (with K=3). Each bar graph shows the prediction result for the averaged classifier and is composed of the probability of prediction of each true class. The classifier was tested using a 5-folds cross validation. This test was done 50 times on a random order data set.
